# Sane or Insane

**Published:** 1830-04-01

**Authors:** 


### XXXIV.

### Sane or Insane.

From a long and sensible Letter in a Scotch periodical, which we know to be written by a physician of great talent in the Modern Athens, we have made some extracts, as we are sure they will be read with interest in this country.

" Regarding the question of the sanity of a fellow-creature as one of the most sacred and important to the decision of which a medical man can be called, and as that on which of all others, it behoves him most to guard himself from the bias of feeling and of prejudice, we cannot but regret the bitterness of spirit under which the London Journals have been writing; for however easy and popular the task of exciting public clamour may be, it is certainly not the way to forward the cause, either of humanity or science. Instead of doing good, which we are willing to believe is their object, by dropping out of sight, as in the present instance, the essential facts and principles from the examination of which alone truth could be elicited and errors exposed, they give countenance and strength to existing prejudices; and by the obloquy thus thrown upon a whole class of the profession, they help to prevent men of high talent, honourable principle, and extensive acquirements, from devoting themselves to a department of medical science in which these qualities would be of the highest value. They do harm also by throwing difficulties in the way of obtaining conscientious testimony on future occasions, and for want of which a *real* lunatic may, under the influence of selfish and designing men, be allowed to go on squandering to the last farthing, and to bring himself and family to misery, starvation, and disease.

Differing entirely, then as we do, from our London brethren in our mode of treating the evil, we heartily concur with them as to its existence and magnitude. It cannot, in truth, be denied, generally speaking, that medical men are very little acquainted with the nature of insanity; and that therefore it becomes them to speak with diffidence on every subject connected with diseased mind. Neither can it be denied that reason and humanity call aloud for the revision of that unjust and barbarous law, which authorises the confinement of a fellow creature on the simple certificate of any two men, however ignorant or dishonest, and in whatever rank of the profession they may be; but still we cannot help thinking, that an ordinary journalist, who has avowedly paid *no* attention to, and knows *nothing* more about mental derangement, than any ordinary observer of human nature, would more effectually bring about a reform by calmly though earnestly pointing out and soliciting attention to apparent deficiencies in the evidence; to sources of error or obscurity; or to the causes of conflicting testimony; by, in short, throwing all the light upon a difficult subject which his talents and acquirements may enable him to do, than by following the opposite plan of rashly dealing out judgment and condemnation on a class of men, who, whatever their faults may be, have paid at least *some* attention to the subject, and know at least *something* more about it than himself; and who, whether we consider the talent and qualifications required for the proper fulfilment of their duties, or the deep importance of the cases entrusted to their care, ought as a body to be raised rather than depreciated in public estimation. But leaving this part of the subject, we have still some remarks to offer on the *principle* lying at the root of the whole inquiry, and not yet, so far as we are aware, sufficiently brought forward.

Without pretending, at this distance, to decide whether Mr. Davies was really sane or insane, we are inclined to suspect, from the published accounts, that, as is usual in most disputes, both parties were somewhat in the wrong; and that, while the newspaper writers erred in holding him to be of perfectly sound mind, the *Mad Doctors* erred in not having given a sufficiently explicit or correct view of the doctrine of insanity. It is one thing, for instance, to determine that a man's mind is in a state of disease, but it is another and very different matter *to determine to what extent the affection has proceeded, and whether it involves only one, or a few, or the whole of the mental powers.* In the case of the stomach, for instance, we can if asked the question, say very certainly, even in a slight disturbance of its function, that it *is* in a morbid condition; but that is very far from necessarily implying that it *cannot* digest food at all, or that digestion is *entirely* deranged. In like manner, abstractly speaking, some of the manifestations of the mind may be positively deranged, but still the patient be competent to the ordinary affairs of life. For insanity is not a specific state, always marked out by well defined lines, which, when it occurs, necessarily unfits a person for mingling in society and in business with his fellow men; but, like affections of other organs, it is a morbid state which may manifest itself in every possible degree, from the most obscure to the most striking departure from mental health. Every body knows, for instance, and the Mad Doctors as well as the rest, that an individual may be *palpably and incurably insane* on all subjects hingeing upon one or two faculties of the mind, and yet be perfectly rational and sound on all others, and that in all matters of thought or of business, which do not touch upon that point, he may continue for years, and even for the remainder of a long life, to display as much shrewdness, prudence, and good sense as nine out of ten of those who never had the fear of a strait-waistcoat before their eyes, and every one conversant with the insane is aware that in practice every possible gradation is to be met with, from an isolated affection like the above, to one involving *all* the faculties of the mind. And consequently the true problem to be resolved, where the rights of liberty and of property are concerned, is not so much whether mental derangement exist, but *whether it has extended so far as to deprive the individual of the power of sound judgment in his own affairs.* Numerous cases, indeed, exist around us of partial affections of the mind which do not interfere in any marked degree with the business habits of the patient, and in which, therefore, it would be the height of cruelty and injustice to deprive him of civil or moral liberty, but in which, at the same time, every conscientious physician, if judicially examined on the abstract question of the existence or non-existence of insanity, would be obliged to answer in the affirmative. Many circumstances indicate this to have been the state of Davies. Some of the witnesses prove that he entertained the most extravagant notions of his own powers and importance, and that he habitually boasted of receiving illumination from Heaven, of being the Son of God, and of being under the special charge of supernatural beings, &c. It is also proved that he was frequently flighty, wild, and incoherent, all of which symptoms might arise from morbid excitement of the single feeling of self-esteem, without the other faculties necessarily participating in the disease. And accordingly we have other witnesses, who were in the habit of transacting business with Mr. Davies, giving it as their decided opinion that he was *not* insane, because "they had taken instructions from him on business, and never had met with a client who better understood his own affairs." Keeping the above distinction in view, we can see no difficulty in believing that Dr. Burrows and his brethren, in saying *on oath* that his mind was *not* sound, were giving not only most conscientious but most true testimony; and that the jury and journalists, *holding competency to business as equivalent to Sanity*, were equally conscientious and correct in pronouncing him to be sane. But if this be the true solution of the contradictory opinions laid before the world, it shows how careful we ought to be in understanding each other's meaning; lest, like the two knights of the olden time, we come to blows about the colour of the shield, when, if each had looked at the other side he would have seen that his opponent was right as well as himself.

There is another condition involving all the faculties of the mind, which may give rise to conscientious difference of opinion, and in which the same distinction ought to be observed. It occurs chiefly in persons of a highly excitable and irritable temperament; who, from trifling causes, are carried away by trains of thinking or idiosyncrasies of feeling, which less susceptible persons experience only after a succession of the most powerful impressions. Persons so constituted pass years of their lives apparently on the verge of insanity, without its ever becoming decided, unless a hereditary predisposition exist, in which case they generally sooner or later lapse into lunacy. In the mean time, however, they are remarkable for unequal spirits, for doing odd things and manifesting strange feelings, but, upon the whole, they conduct themselves so much like other people, that although every one remarks that they have their peculiarities, few will venture to pronounce them *insane.* But in such cases, when the transition to insanity does occur, it is so gradual, that the most experienced physician, even after maturest examination, is often left in doubt as to the extent to which the disease has proceeded; and while he feels that the individual is not in a condition to be left entirely to his own guidance, he is at the same time conscious that he retains too much soundness of mind not to be injured by the premature interference either of friends, of doctors, or of lawyers.

The point of difficulty for the physician, therefore, and that for the solution of which we would most ardently long for the assistance of an intelligent jury, is to determine, not the mere existence of a mental affection, but *the limit at which that affection begins to deprive the individual of the power of proper self-direction, and at which, therefore, it becomes the duty of the law and of the friends to step in for his protection.* The right solution of this problem is no easy task, for it requires in the Jurors not only clearness of perception and soundness of judgment, but a knowledge of human nature, and an acquaintance with the general functions of the body, and with the previous habits and constitution of the suspected lunatic, which unhappily, under our imperfect systems of general education, very few persons are found to possess. And it is in vain to seek for any general rule to help us out of the difficulty, for every human being presents so many points of difference in mind and in body, and in the external circumstances modifying both, that every new case requires the same partial examination, the same careful analysis, and the same accurate consideration of all the attendant phenomena as the first that ever occurred to us, and he who, disregarding all these conditions, hastens to form his opinion from the application of general rules, will inevitably fall into error, and be the cause of much misery to those who confide in him. But not to encroach too far on the patience of our readers, we shall conclude, trusting that we have said enough to satisfy our brethren that they have been too rash and reckless in exciting a clamour against the profession, and that the question in debate, is really not so easy of solution as they seem to have imagined."

---

